# Adverse Fetal and Neonatal Outcomes Associated with a Life-Long High Fat Diet: Role of Altered Development of the Placental Vasculature

**DOI:** 10.1371/journal.pone.0033370

**Published:** 2012-03-19

**Authors:** Emily K. Hayes, Anna Lechowicz, Jim J. Petrik, Yaryna Storozhuk, Sabrina Paez-Parent, Qin Dai, Imtiaz A. Samjoo, Margaret Mansell, Andree Gruslin, Alison C. Holloway, Sandeep Raha

**Affiliations:** 1 Department of Pediatrics, McMaster University, Hamilton, Ontario, Canada; 2 Graduate Program in Medical Sciences, McMaster University, Hamilton, Ontario, Canada; 3 Department of Biomedical Sciences, University of Guelph, Guelph, Ontario, Canada; 4 Division of Maternal Fetal Medicine, Department of Obstetrics and Gynecology, Ottawa Hospital Research Institute, University of Ottawa, Ottawa, Ontario, Canada; 5 Department of Cellular and Molecular Medicine, Ottawa Hospital Research Institute, University of Ottawa, Ottawa, Ontario, Canada; 6 Department of Obstetrics and Gynecology, McMaster University, Hamilton, Ontario, Canada; Erasmus University Rotterdam, The Netherlands

## Abstract

Maternal obesity results in a number of obstetrical and fetal complications with both immediate and long-term consequences. The increased prevalence of obesity has resulted in increasing numbers of women of reproductive age in this high-risk group. Since many of these obese women have been subjected to hypercaloric diets from early childhood we have developed a rodent model of life-long maternal obesity to more clearly understand the mechanisms that contribute to adverse pregnancy outcomes in obese women. Female Sprague Dawley rats were fed a control diet (CON - 16% of calories from fat) or high fat diet (HF - 45% of calories from fat) from 3 to 19 weeks of age. Prior to pregnancy HF-fed dams exhibited significant increases in body fat, serum leptin and triglycerides. A subset of dams was sacrificed at gestational day 15 to evaluate fetal and placental development. The remaining animals were allowed to deliver normally. HF-fed dams exhibited a more than 3-fold increase in fetal death and decreased neonatal survival. These outcomes were associated with altered vascular development in the placenta, as well as increased hypoxia in the labyrinth. We propose that the altered placental vasculature may result in reduced oxygenation of the fetal tissues contributing to premature demise and poor neonatal survival.

## Introduction

Data from the Centre for Disease Control indicate that in 2008, almost 20% of children between ages 6–11 were considered obese, and many of the females in this group are likely to remain obese through their childbearing years [1]. In fact, epidemiological studies report that 15–20% of women of reproductive age in Canada, US, Australia and the UK are now clinically obese (BMI>30 kg/m^2^) [2]–[4]. With obesity rates on the rise, it is clear that the consequences of life-long obesity on pregnancy outcomes need to be addressed. There is compelling evidence that obese women are at increased risk for many pregnancy-related complications to their own health, such as gestational diabetes and preeclampsia [5]–[7]. In addition to maternal health issues, there is also a considerable increase in the risk of fetal complications such as spontaneous abortions, fetal asphyxia, and stillbirth [8], as well as increased risk of delivery of both small for gestational age (SGA) and large for gestational age (LGA) babies [2], [9], [10]. Pre-gravid obesity also contributes to reduced fertility in women [6], [11], [12]. This may be due to disruptions in oocyte function [13] or a chronic state of inflammation associated with obesity [14], which may contribute to impaired implantation of the blastocyst [15].

While the mechanisms behind the obesity-mediated reduction in fecundity are not clearly understood, current speculation suggests that altered levels of cytokines and adipokines, as well as hormones such as insulin, may impact ovulation and implantation (reviewed in [16]). Maternal obesity is also associated with a number of metabolic disturbances such as insulin resistance, elevated serum triglycerides, and increased blood pressure (reviewed in [17]). In addition to these systemic effects, obesity has also been associated with tissue specific changes in mitochondrial function and elevated production of reactive oxygen species (ROS) leading to increased oxidative stress [18], [19].

Some of these changes associated with maternal obesity may lead to alterations in placental development or function. Of critical importance to proper placental development is the establishment of the vascular architecture between maternal and fetal circulatory systems. Functional deficiencies at this interface can contribute to intrauterine growth restriction (IUGR) and pre-eclampsia (reviewed in [20]), as well as a significant increase in premature fetal demise [6], [21], [22]. While previous epidemiological studies have linked increased BMI with placental dysfunction and adverse obstetrical outcomes [2], [23] the mechanisms for such associations have not been fully elucidated.

The majority of animal models that attempt to address the consequences of maternal obesity focus on postnatal outcomes [24] or subject the dams to short-term high fat (HF) diets just prior to pregnancy or during gestation [25], [26]. This has resulted in a relative paucity of data regarding the consequences of long-term maternal obesity on placental function and fetal development. We have developed a rodent model which addresses the consequences of life-long obesity on the development of the placenta. Our model suggests that a life-long high fat diet results in altered vascularization of the placenta and this has adverse consequences for fetal survival. We observe increased numbers of stillbirths and reduced weight at birth, as well as reduced neonatal survival. These poor fetal and neonatal outcomes may be due to increased placental hypoxia and a trend towards increased oxidative stress as a consequence of altered development of the placental vasculature.

## Results

### HF-diet results in increases in the biomarkers associated with obesity

**Figure 1:**
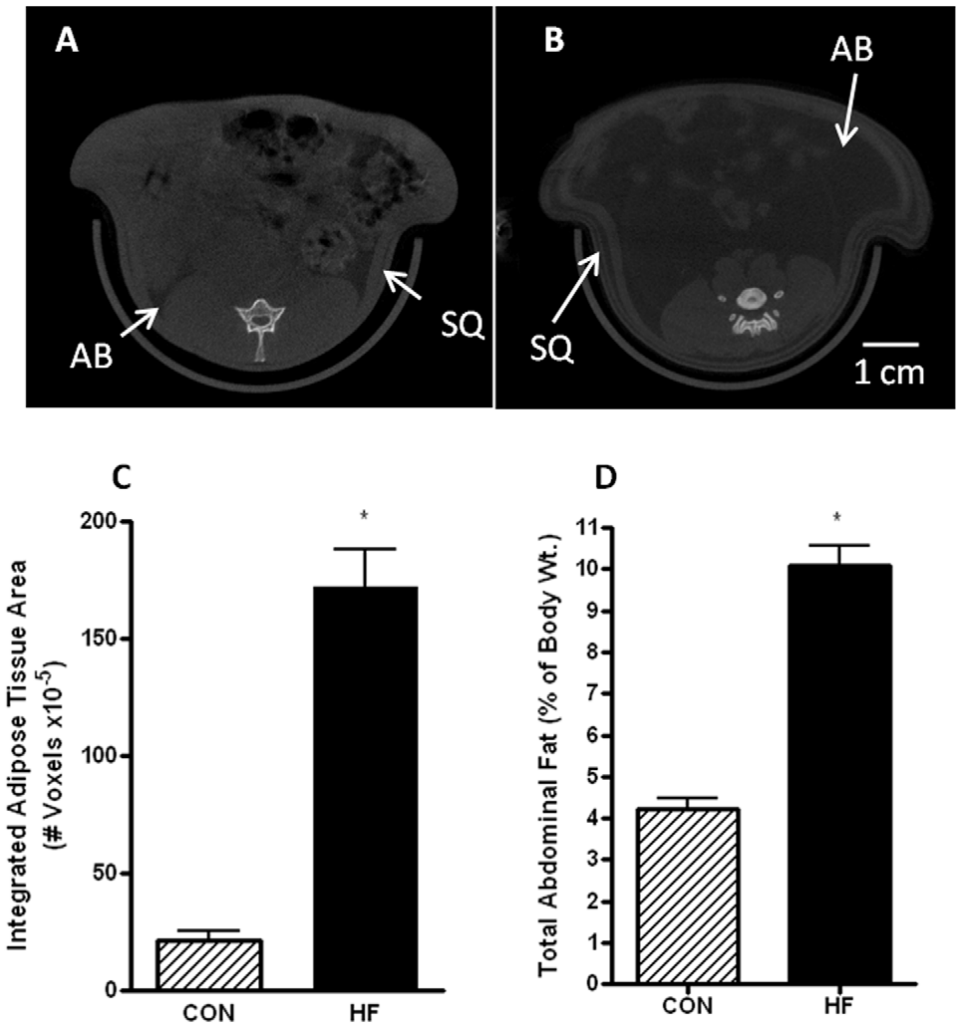
CT analysis demonstrates that a HF-diet leads to increased subcutaneous and abdominal fat. Fasted rats were anesthetized and subjected to CT scanning. A. Representative CT-scan of a CON-fed dam; B. Representative CT-scan of a HF-fed dam. Adipose tissue is characterized by a lower intensity signal (darker regions on the image). The area occupied by the abdominal fat (AB) and subcutaneous fat (SQ) are indicated; scale bar = 1 cm. C. Adipose tissue absorption in the area between the bottom of the lungs and the top of the sacroiliac joint was calculated with Amira software using attenuation thresholds of −150 to −400 Hounsfield units. The values represent mean ± SEM. * p<0.01; n = 7 in each group. D. The mass of total abdominal adipose tissue during pregnancy (GD15) was also quantified and expressed as a percentage of body weight. The total pool includes gonadal, retroperitoneal and mesenteric fat. Values represent mean ± SEM; *p<0.01, n≥18 in each group.

HF-fed dams were 22% heavier by 19 weeks of age (Figure S1) and had 3-fold greater levels of serum leptin compared CON-fed dams (Table 1). To assess the body fat composition in dams prior to pregnancy, CT scans (n = 7 in each group) were performed at 19 weeks of age (Figures 1A and 1B). The total body fat content for the area between the bottom of the lungs and the top of the sacroiliac joint was approximately 7 fold higher in the HF-fed dams as compared to the CON-fed dams (Figure 1C). In addition, abdominal fat pad weight during pregnancy (GD15) was found to be 2.5-fold greater in HF-fed animals (10.08±0.49% vs. 4.24±0.25% of total body weight; Figure 1D).

**Table 1:** Pre-pregnancy metabolic parameters of fasted dams.

Outcome measure	CON-fed Dams	HF-fed Dams	P
Fasting Glucose (mmol/L)	4.9±0.3	5.7±0.2	0.03*
Fasting Insulin (ng/mL)	1.0±0.2	1.2±0.3	0.64
Glucose AUC	290.1±47.1	247.7±55.2	0.56
Insulin AUC	150±4.4	167±7.0	0.01*
HOMA-IR	0.11±0.02	0.11±0.05	0.79
Triglyceride (mg/dL)	72.5±13.9	131.8±19.0	0.02*
NEFA(mmol/L)	0.5±0.1	0.6±0.1	0.41
Total Cholesterol (mg/dL)	110.2±7.3	106.0±7.8	0.34
Leptin	3.55±0.51	12.72±1.69	0.0001*

Values are mean ± SEM,

* significantly different between CON-fed and HF-fed dams.

In order to determine if increased body weight prior to pregnancy was associated with pre-pregnancy dysglycemia, we determined fasting glucose, insulin and HOMA-IR levels at 17 weeks of age (Table 1). Fasted glucose levels were significantly elevated in HF-fed dams prior to mating (CON: 4.9±0.3 mmol/L vs. HF: 5.7±0.2 mmol/L, p<0.05); however, fasted insulin levels and HOMA-IR were not significantly different. Interestingly, while the oral glucose tolerance test in these animals did not exhibit a significant difference between the two groups of dams, the insulin tolerance test demonstrated that HF-fed dams had a significantly greater area under the curve (AUC; Table 1) compared to CON-fed dams.

Prior to mating, HF-fed dams had significantly higher levels of fasting serum triglycerides (1.8 fold higher than CON-fed dams). Leptin levels were 3.5 fold greater in the HF-fed dams as compared to the CON-fed dams. Circulating non-esterified fatty acids (NEFA) and cholesterol levels were not different between the two groups.

### Elevated gestational blood pressure in HF-fed dams

**Figure 2:**
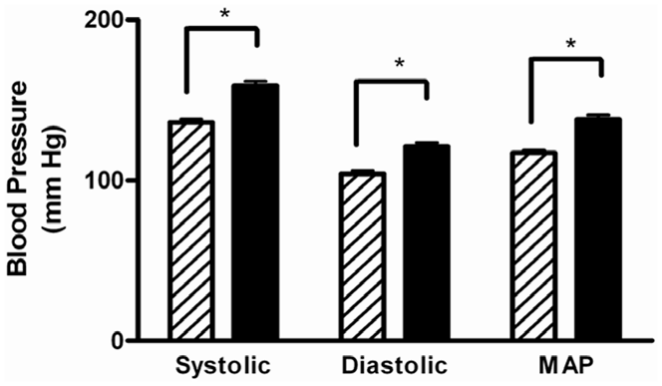
Arterial Blood Pressure in elevated in obese dams at GD 15. Blood pressure was determined at GD15 using tail cuffs. Animals were gently restrained in cages warmed to 37°C using a heating pad. The hatched bars represent CON-fed animals, while the solid black bars represent HF-fed animals. Values are mean ± SEM; ^*^p<0.05, n≥23 in each group. The animals underwent 5 acclimatization cycles and 20 measurement cycles. The reported values are the mean of these 20 cycles.

HF-fed dams had significantly higher systolic, diastolic and mean arterial pressure (MAP) at GD15 relative to normal weight dams (Figure 2). The difference in the MAP between HF-fed and CON-fed animals, was 21 mmHg (138±2.8 vs 117±1.7 mmHg). While the HF-dams did exhibit a higher blood pressure prior to pregnancy they also demonstrated a greater increase in their blood pressure over the course of their pregnancy as compared to the CON-fed dams (Figure S2). The greatest degree of difference between the two groups was evident between GD15 and GD20.

### Life-long HF-diet affects obstetrical outcomes

A HF-diet for 4 months prior to mating impacted a number of obstetrical and fetal outcomes (Table 2). Time to copulation was significantly longer for HF-fed dams relative to CON-fed dams (3.80±0.41 vs. 2.59±0.39 days for CON-fed dams). Furthermore, the mating success for HF-fed dams was reduced by 32.4%, while the fertility index for this group was 22.1% lower. A significantly higher proportion of the offspring of HF-dams were small for gestational age (CON: 0.7% - 3/383 pups vs. HF: 4.7% -11/234 total pups). Most interestingly, there was a dramatic shift in the male/female ratio for HF-fed dams. The sex ratio (M/F) for CON-fed dams was 1.19±0.14; in contrast, the ratio for HF-fed dams was 0.67±0.11 (p = 0.008) indicating that significantly fewer males were born to this group of mothers.

**Table 2:** Obstetrical outcomes of CON and HF fed dams.

Obstetrical Outcome	CON-fed Dams	HF-Fed Dams	P
Body Weight Prior to Pregnancy	303±5.9	412±11.9	0.001*
Average time to copulation (d)	2.59±0.39	3.80±0.41	0.03*
Mating success (%)	100	67.6	0.004*
Fertility Index (%)	73.8	51.7	0.03*
Live Birth Index	98.9±0.6	87.7±4.9	0.03*
LGA	11/383	0/234	0.003*
SGA	3/383	11/234	0.009*
Sex Ratio (M/F)	1.19±0.14	0.67±0.11	0.008*

Values are mean ± SEM,

* significantly different between CON-fed and HF-fed dams.

### Maternal obesity leads to compromised fetal growth and poor neonatal health

**Figure 3:**
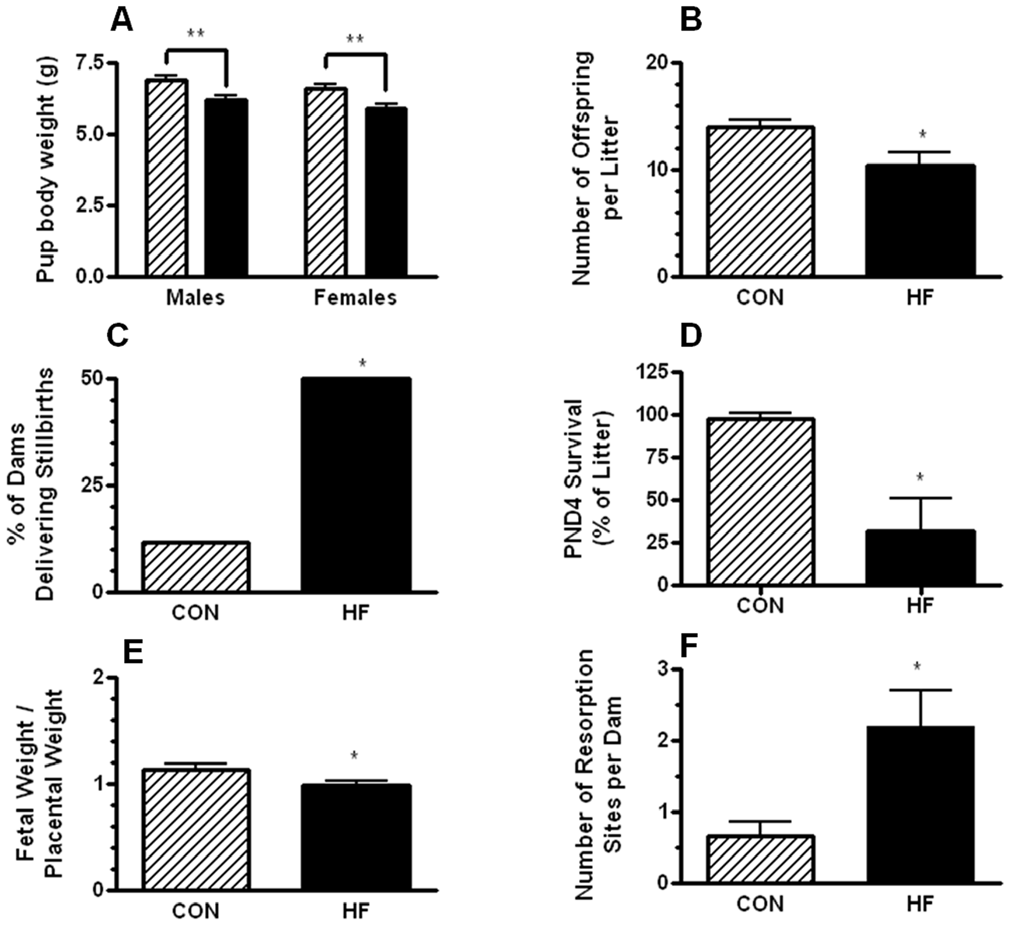
Neonatal health outcomes for pups born to CON or HF-fed dams. A. The body weight of offspring of CON-fed (hatched bar) or HF-fed (solid black bar) dams on postnatal day 1 (PND1). B. The average number of pups per litter born to CON-fed or HF-fed dams. C. The percentage of CON-fed or HF-fed dams giving birth to at least one stillborn pup. D. The percentage of the total number of live pups in the litter that survived to PND4. E. The fetal/placental weight ratio was calculated for CON-fed and HF-fed dams. F. The average number of resorption sites for CON-fed and HF-fed dams at GD15. Values represent mean ± SEM; ^*^p<0.05; n≥12 dams per group.

Life-long maternal obesity impacts fetal growth and development. Obese dams not only had 30% fewer pups per litter (Figure 3B) but individual pup weights, both males and females, were reduced by 12% (Figure 3A). Even more striking was the significant increase in the number of HF-fed dams that gave birth to stillborn pups. 45% of the HF-fed dams compared to only 10% of CON-fed dams delivered stillborn pups (Figure 3C). Furthermore, the live pups born to HF-fed dams had reduced survival to post-natal day 4, an indicator of poor neonatal health [27]. Indeed, only 25% of the pups born to HF-fed dams survived past PND4 (Figure 3D).

*In utero* fetal development was also affected by maternal obesity. Evidence of premature fetal demise in the HF-fed dams was indicated by an almost 3-fold increase in the number of uterine absorption sites (Figure 3F). Furthermore, the fetal to placental weight ratio also exhibited a 12% reduction in this group of dams (p<0.05) (Figure 3E).

### The placenta of HF-fed dams are characterized by altered vascular development

**Figure 4:**
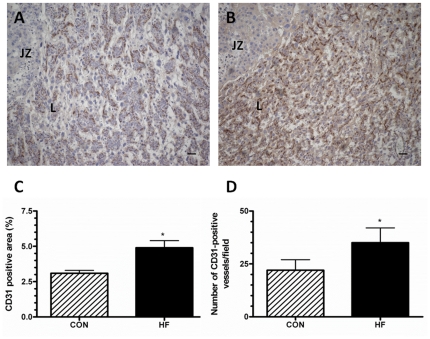
The labyrinth layers of placentas from HF-fed dams exhibit increased expression of endothelial cell markers at GD15. Representative images, acquired at 100× magnification, of GD15 placenta from CON-fed (A) and HF-fed dams (B) immunostained with CD31 antibody are shown. Scale bar = 50 µm; the labyrinth (L) and junctional zone (JZ) are indicated for reference. Four distinct regions from each histological section were quantified and averaged in determining percent immunopositive area. Images from an individual dam represent a single statistical unit. C. The percentage of area immunopositive for CD31 in the labyrinth of GD15 placenta based on the analysis of cross sections from CON-fed and HF-fed dams sacrificed at GD15. D. The number of blood vessels per field for CON-fed or HF-fed dams at GD15. All values mean ± SEM, ^*^p<0.05; n = 5 dams for each group.

**Figure 5:**
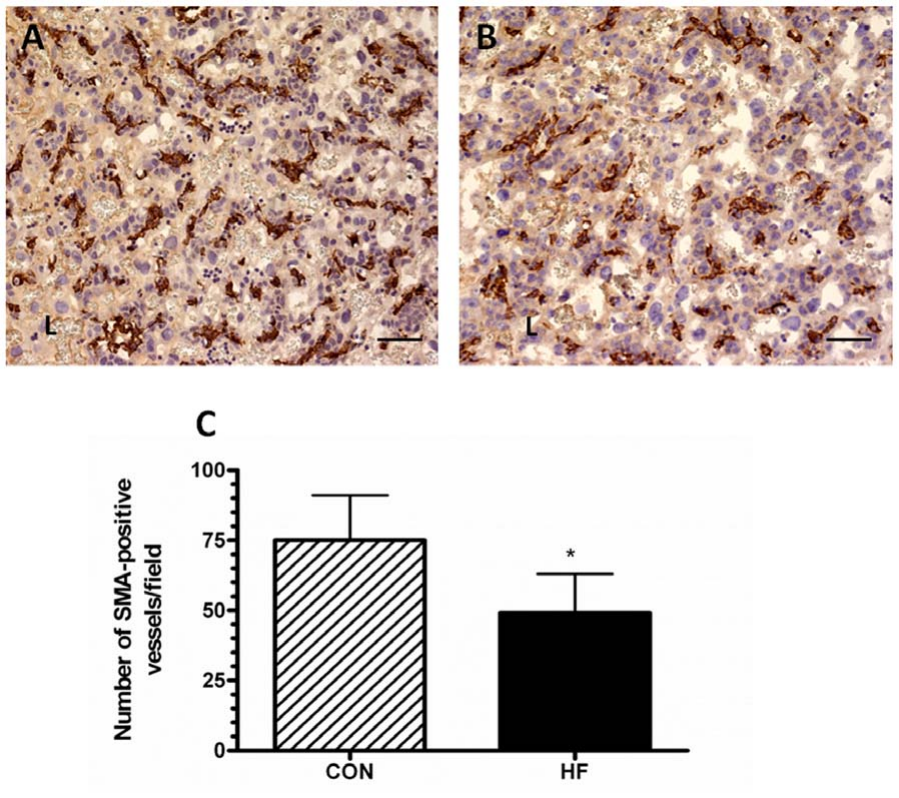
The labyrinth layers of placentas from HF-fed dams exhibit fewer SMA positive blood vessels at GD15. Representative images, acquired at 200× magnification, of GD 15 placenta from CON-fed (A) and HF-fed dams (B) immunostained with an antibody to SMA are shown. The SMA positive area within the labyrinth (L) of each group of animals was quantified. C. The average number of vessels per field of view staining positive for smooth muscle actin (SMA) in placentas from CON-fed and HF-fed dams. D. All values mean ± SEM; ^*^p<0.05, n = 5 dams for each group.

Maternal obesity affected blood vessel density and maturity (Figure 4 and 5) in the labyrinth. There was an almost 2 fold increase in the CD31 positive area, an endothelial cell marker, in the placenta of HF-fed dams compared to controls (Figure 4C). Accordingly, the blood vessel density within the placenta (number of CD31-positive blood vessels per field) was higher (Figure 4D). However, there was a 30% decrease in the number of blood vessels that stained positive for smooth muscle actin (SMA), a marker expressed by vascular smooth muscle cells and found in mature blood vessels (Figures 5).

### The placentas of HF-fed dams are characterized by reduced tissue oxygenation

**Figure 6:**
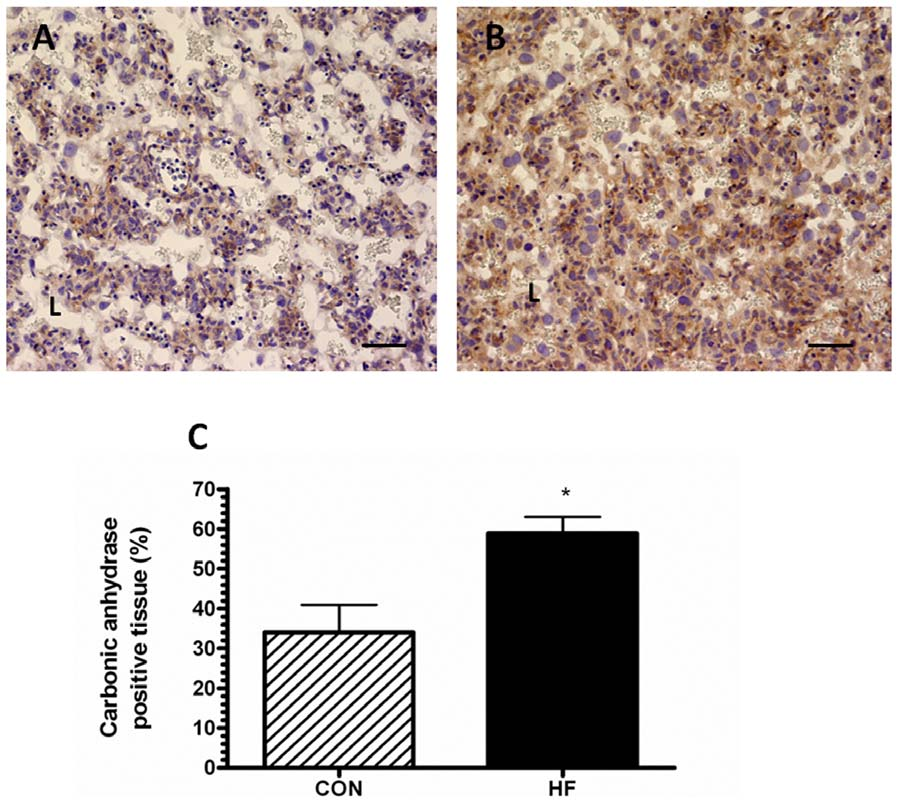
The labyrinth layers of placentas from HF-fed dams exhibit increased levels of carbonic anhydrase staining at GD15. Representative images, acquired at 200× magnification, of GD 15 placenta from CON-fed (A) and HF-fed dams (B) immunostained with an antibody to carbonic anhydrase are shown. C. The average staining intensity in placentas from CON-fed and HF-fed dams was calculated. All values mean ± SEM, ^*^p<0.05; n = 5 dams for each group.

The levels of carbonic anhydrase within the placenta were evaluated as an indicator of tissue hypoxia [28]. Carbonic anhydrase-immunopositive tissue in HF placenta was increased by 47% over CON placenta (Figure 6A–C).

**Figure 7:**
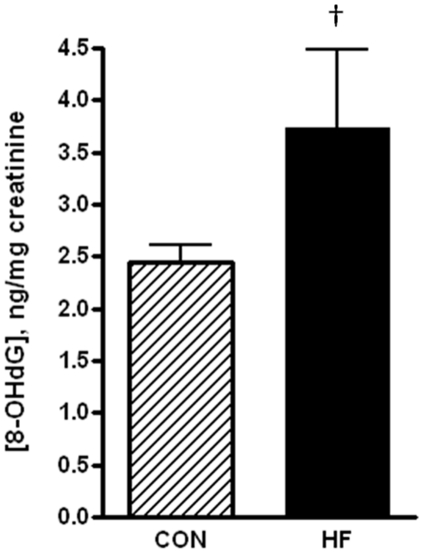
8-hydroxy 2-deoxyguanosine, a marker of systemic oxidative damage, is not significantly increased in the HF-dams. Levels of 8-OH-2-dG were quantified in urine collected from GD15 CON-fed and HF-fed dams at sacrifice using a competitive EIA kit. 8-OH2-dG was normalized to the concentration of creatinine in urine. All values mean ± SEM, n = 15 for CON and n = 10 for HF-fed dams. ^†^p<0.1 by one tailed Students T-test.

**Figure 8:**
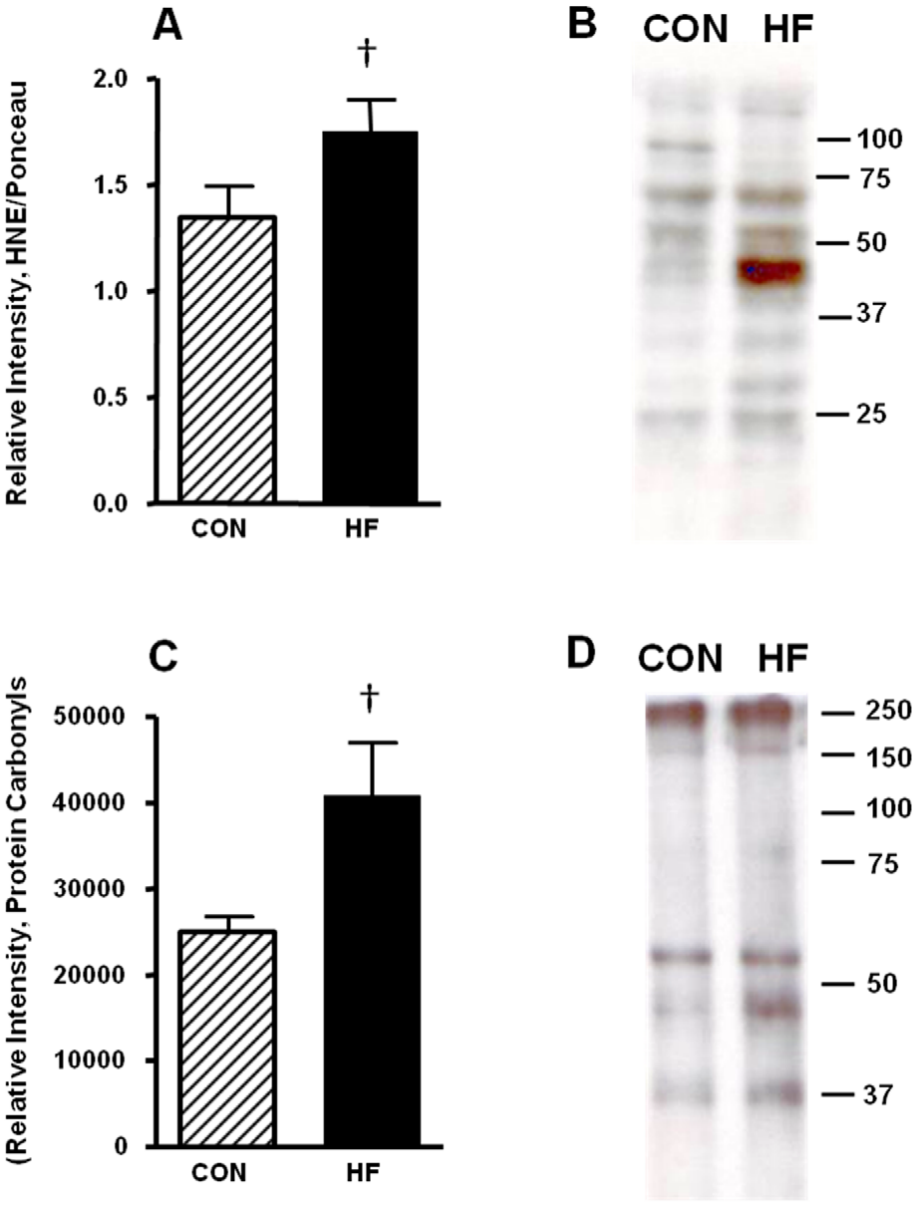
Markers of tissue specific oxidative damage are not significantly increased in placentas from HF-fed dams at GD15. A. 10 µg of total whole placental homogenate was separated on a 12.5% SDS-PAGE and subjected to Western blot analysis. The average content of 4-HNE was normalized to total protein (using Ponceau-S staining) in CON (hatched bars) or HF-fed (solid black bars) dams. B. Representative lanes containing 10 µg placental homogenates, developed using the 4-HNE monoclonal antibody. C. The relative content of protein carbonyls was quantified using a polyclonal antibody directed towards 2,4-dinitrophenylhydrazine (DNPH). This quantification was carried out using 5 µg of placental homogenate enriched for mitochondria. D. Representative lanes containing 5 µg of protein, enriched for mitochondria, prepared from CON or HF-fed dam placenta were separated on a 12.5% SDS-PAGE and developed using the anti DNPH polyclonal antibody. Values represent mean ± SEM; ^†^p<0.10, n = 6 per group.

Since hypoxia is known to increase oxidative damage in the placenta [29] we assessed whether there was a change in the level of systemic oxidative damage in obese pregnant dams by quantifying urinary 8-hydroxy- 2-deoxyguanosine (8OH-2-dG). This is a well characterized marker of oxidative damage, and its increase in obesity has been reported in both humans [30] and rodents [31]; therefore we carried out a one-tailed Students T-test with respect to the significance of this data. Urinary 8OH-2-dG levels in the HF-fed animals were 25% higher relative to the CON-fed dams; however this did not reach statistical significance (p<0.1) (Figure 7). There was also an increase in the mean levels of 4-HNE (p<0.1) in whole placental homogenates of GD15 HF-fed dams (Figure 8A and B). Interestingly, a mitochondrially enriched fraction isolated from the placentas of HF-fed also exhibited increases in the levels of protein carbonyls, a marker of oxidative protein damage (Figure 8C and D; p<0.1). While neither of these markers of oxidative damage were statistically different in the placenta of CON-fed vs HF-fed animals, the mean levels are consistently higher in the HF-fed dams.

## Discussion

The effects of maternal obesity on pregnancy outcomes in humans are well documented. Despite the plethora of epidemiological data surrounding the consequences of maternal obesity, the mechanistic understanding of the cellular signaling that mediates poor health outcomes for both mother and fetus are far from clear. These adverse outcomes include metabolic complications in the offspring [32], obstetrical complications in the mother [9], [21] and abnormal fetal growth [22], [33]. The lack of a mechanistic understanding of these processes may be due, in part, to the difficulty of developing animal models that emulate all the complications of obesity during pregnancy in humans. The majority of rodent models designed to address the consequences of maternal obesity have been focused on understanding metabolic reprogramming in offspring and have not focused on factors responsible for premature fetal demise and poor neonatal health.

Our rodent model of life-long maternal obesity is characterized by the well accepted hallmarks associated with obesity such as elevated serum leptin and triglycerides and increased body fat. An evaluation of glucose homeostasis prior to pregnancy demonstrated that the HF-fed dams had a small but significant increase in their fasting glucose levels and were insulin resistant (i.e., had an increased area under the curve following an insulin challenge). This is to be expected with such obese animals. However the results of the glucose tolerance test did not prove to be significantly different between the groups. This suggests that the pancreas has been able to modulate insulin secretion to maintain glucose homeostasis and these animals have not yet developed overt type 2 diabetes mellitus. Taken together the data suggest that there is partial loss of glycemic control in the HF-fed animals which may become aggravated during pregnancy. We did not conduct OGTT measurements during pregnancy due to the possibility compromising fetal health.

The model also demonstrates many of the fetal and maternal complications seen in human pregnancies associated with obesity; increased blood pressure during pregnancy, reduced fetal growth, and increased fetal/neonatal demise [22], [34]. In addition to the parallels in fetal outcomes, we also observed obstetrical complications associated with obesity in reproductive age women such as reduced fertility [6]. Interestingly, the majority of epidemiological studies report that obese women are at significantly greater risk for delivering large for gestational age (LGA) babies, yet there is also an elevated risk for small for gestational age (SGA) babies which has also been noted in humans, primates and rodents [10], [34], [35]. An evaluation of singleton pregnancies between 1978–1997 in Missouri (over 310,000 pregnancies) found that SGA babies born to obese mothers were at significantly greater risk for neonatal death [36] compared to LGA babies born to this group. Furthermore, the risk of premature death for these neonates increased with degree of their mother's obesity [24], [26]. Since the mechanisms for such phenomenon are not well understood it underscores the importance for developing an animal model for elucidating the pathways leading to premature fetal demise and poor neonatal health.

Reduced fetal growth and premature fetal demise, as a consequence of obesity during pregnancy, may implicate placental dysfunction. Similar outcomes have been connected with placental dysfunction in preeclampsia and IUGR [38]. While higher rates of preeclampsia are associated with obesity [39], it has been argued that this is a uniquely human condition and may not be accurately emulated in rodent models [40]. Two recent studies have also reported poor fetal growth in association with maternal obesity, but have not evaluated premature fetal demise in their respective models. Grove and colleagues [41] demonstrated that a life-long high-fat diet in Japanese macaques resulted in fetal growth retardation. Similarly, in a rodent model, Akyol et al. showed that Sprague-Dawley dams fed a cafeteria diet prior to and during pregnancy had lower fetal and placental weights, as well as a lower fetal∶placental weight ratio at GD20 of pregnancy [35]. In agreement with these observations, the birth weight of the pups from our cohort of HF-fed dams was reduced by 12%, and we also observed a decreased fetal∶placental weight ratio at GD15. However, this growth retardation was not as profound as has been reported in rodent models of chemically induced pre-eclampsia (more than a 30% decrease in pup birth weight) [38].

The current model of maternal obesity is also characterized by a significant increase in the number of HF-fed dams delivering stillborn pups, as well as an increase in the number of fetal resorption sites; further evidence of premature uterine demise. Epidemiological data suggest that the mechanisms behind a large percentage of reported stillbirths are not well understood [8]. Our rodent model clearly points to a role for maternal obesity-associated placental dysfunction in increasing the risk of stillbirths and supports the existing epidemiological data [12].

In humans, epidemiological data has been used to suggest a correlation between metabolic syndrome and placental dysfunction [23]. It is important to note that while the temporal relationships in placentation differ between rodents and humans, the early processes leading to the establishment of the maternal-fetal interface are common, making rats a good model for placental studies [37].

In order to understand why the fetuses of HF-fed dams failed to thrive, we analyzed the histomorphology of the placenta at mid-gestation (GD15). We observed an increase in CD31 staining in the labyrinth of placentas from HF-fed dams indicating the presence of greater numbers of blood vessels, an observation supported by recent data which has demonstrated that vascularization is also dysregulated in the placental cotyledons of the obese ewe [35]. Interestingly, the increase in blood vessel density, observed in our model, was inversely proportional to the staining with SMA, an indicator of blood vessel maturity [42]. A reduction in the presence of SMA staining surrounding endothelial cells in the labyrinth has been associated with increased gestational blood pressure and reduced fetal growth in a mouse model of preeclampsia [43].

Together this suggests that placenta of HF-fed dams may have significant deficits in the processes involved with blood vessel maturation. The poor development of the placental vasculature in the placenta of obese dams may result in reduced blood flow to the placenta, an observation which is consistent with the evidence of hypoxia in the placenta of HF-fed dams. In humans, changes in vascularization and hypoxia in the placenta are associated with several disorders of pregnancy, including IUGR, SGA fetuses and preeclampsia [44]. Hypoxia is known to be a signal for the production of VEGF [45] which may trigger increased angiogenesis and would be consistent with the increased CD31 staining observed in the placenta's of our HF-fed dams.

Hypoxia is also associated with increased oxidative stress [29] which in turn can result in significant placental pathology [46]. Furthermore, systemic increases in the markers of oxidative stress have also been reported in obese humans [30] as well as rodents [31]. In general, oxidative stress results from an imbalance between increased cellular ROS production and cellular antioxidant defenses. If ROS production overwhelms cellular defenses then this can result in either increased cellular damage or adaptive changes mediated by free radical signaling [47]. We therefore assessed the level of oxidative damage in our HF-dams by using several markers of systemic as well as tissue specific oxidative damage. While our HF-fed dams did not exhibit a statistically significant increase in systemic oxidative damage, the mean level of urinary 8OH-2-dG (a marker of oxidative damage to DNA), was higher in this group. In addition to being associated with obesity, increases in 8OH-2-dG in maternal mid-gestational urine has been associated with reduced fetal weight at birth in humans [48]. Oxidative stress results from the production of superoxide radicals from a number of cellular sources, including mitochondria, which ultimately contribute to the formation lipid peroxides, protein carbonyls, or 8OH-2-dG [47], [49]. These represent markers of lipid, protein and DNA damage respectively. The consequence of such increases in ROS are dictated by the reactivity and lifetime of the reactive species (reviewed in [50]). Interestingly, recent work by Myatt and colleagues suggests that the majority of the oxygen free radicals formed in the pre-eclamptic placenta is converted to peroxynitrite and this results in an increase in protein nitration and perhaps a decrease in overall markers of oxidative damage [51]. Our attempts to compare the level of nitrotyrosine modification in the GD15 placental homogenates of CON-fed vs. HF-fed dams demonstrated that while there was an increase in the mean of the level of protein residues modified by nitrotyrosine the values did not reach statistical significance (Figure S3). We also observed a small increase in the mean levels of lipid damage (4-hydroxy-2-noneal) in the placenta of HF-fed dams (p<0.1). We therefore examined whether oxidative damage may be more evident in isolated mitochondria because they are considered to be one of the primary producers of cellular superoxide. Due to the limited diffusion distance of superoxide, the probability of damage proximal to the site of production may be greater. We demonstrate in this study that the mitochondria isolated from whole placenta of HF-fed dams also exhibited increased mean levels of protein damage (protein carbonyls) but these did not reach statistical significance. While we cannot conclude that there is an increase in the level of oxidative stress in the placenta of obese dams, it is interesting to note that three separate markers of oxidative damage all exhibited increased mean levels in the placenta of obese dams. Furthermore, we cannot exclude the absence of oxidative stress because of the possibility that diet may have resulted in increased expression of ROS defense proteins which may attenuate the level of damaging radical species.

The altered vascular development and associated hypoxia in the placentas of HF-fed dams may contribute to poor fetal growth and neonatal survival. Our model demonstrated that the offspring of HF-fed dams exhibited increased neonatal death. A link between reduced fetal growth *in utero* and poor neonatal health has been suggested based on the observation that SGA babies born to obese women exhibit reduced post-natal survival [36]. However, neonatal demise may be the result of a number of perinatal, as well as postnatal factors. For example, epidemiological studies have pinpointed maternal obesity as a predictor of delayed onset of lactation as well as a reduced intention to breastfeed [52], [53]. Recent work by Bautista et al. suggest that maternal obesity, in a rat model, results in reduced breast milk production higher fat and leptin content [54]. Our current experimental design does not allow for the delineation of the extent to which such postnatal factors contribute to the early demise of the neonates.

Taken together our model of life-long maternal obesity in rodents exhibits many of characteristics demonstrated clinically in obese humans including premature fetal demise and reduced fetal growth. Our data is consistent with the view that excess adiposity during pregnancy alters the development of the placental vasculature and this reduces tissue oxygenation. Using examples of undernutrition during pregnancy, it been suggested that the fetus adapts to increased uterine stress and hypoxia by sparing the growth of the brain and reducing body growth [55], [56]. It is possible that this increased uterine stress, as a result of maternal obesity, facilitates the development of defects in the pathways responsible for the proper development of the placental vasculature. In fact, it has been suggested that failure to remodel the spiral arteries may be associated with increased oxidative stress and pregnancy failure in humans [57]. It will therefore be important to delineate the mechanisms by which obesity can affect development of placental vasculature. The elucidation of these pathways will contribute to understanding how maternal obesity affects poor fetal development and premature death.

## Materials and Methods

### Animal protocol and sampling of histological preparations

All animal procedures for this study were approved by the McMaster University Animal Research Ethics Board (Animal Utilization Protocol 07-07-40) in accordance with the guidelines of the Canadian Council of Animal Care. Female Sprague-Dawley rats, aged 21 days (84–100 g), were purchased from Charles River Laboratories (Willmington, MA). Rats were maintained under controlled lighting (12 hr light – dark cycle) and temperature (22°C) with *ad libitum* access to food and water. Dams were randomly assigned to receive either standard rat chow (CON) (16% kcal fat, 3.82 kcal/g; Harlan Teklad, Madison, WI) or a high fat (HF) diet (45% kcal fat, 4.70 kcal/g; Research Diets, New Brunswick, NJ) (Table - S1). Body weights of each dam were monitored biweekly. Dams were maintained on their respective diets for 16 weeks before being mated with age-matched Sprague-Dawley males fed the CON diet. Copulation was confirmed by the presence of sperm in a vaginal flush; the day of copulation was designated gestational day 0. A subset of dams (CON n = 20, HF n = 18) underwent laparotomy at GD15, exposing the uterine cavity with the fetuses. Resorption sites were counted and each fetus and its corresponding placenta were separated and weighed. Placental tissue samples were snap-frozen in liquid nitrogen storage or placed in 10% neutral buffered formalin for immunohistochemical analysis. The remaining dams (CON n = 25, HF n = 20) were allowed to give birth normally.

### Measures of obstetrical outcomes

A number of obstetrical outcomes including average time to copulation (number of days cohabited to achieve a sperm positive vaginal flush), mating success (sperm positive dams/total number of cohabiting pairs), fertility index (number of confirmed pregnancies/sperm positive dams), live birth index (number of live pups/total number of pups for each litter), sex ratio (M∶F) and litter size were determined. We also determined the number of pups that were either large for gestational age (LGA; birth weight greater than 2 standard deviations above the average birth weight of control pups) or small for gestational age (SGA; birthweight less than 2 standard deviations below the average birthweight of control pups).

### CT Scanning and Analysis

To determine the amount of visceral and subcutaneous fat accumulation in the abdominal area, a subset of the animals in each group were fasted for 16 hr at 19 weeks of age (CON = 7 and HF = 7) and CT scanning was carried out using the X-SPECT small animal imaging system (Gamma Medica, Northridge, CA, USA). The midsection of the rat was scanned and the area between the bottom of the lungs and the top of the sacroiliac joint was selected for fat quantification. This area contained approximately 100 slices, each 0.5 mm apart. Densitometric quantification of fat was carried out using Amira software with attenuation thresholds of −450 to −150 Hounsfield units (HU) to select for fat tissue. Air bubbles in the intestines were first removed from the image, and the total fat (subcutaneous+abdominal) was then quantified by integrating all voxels (a 3-dimensional pixel) between −450 and −150 HU.

### Blood pressure analysis

Blood pressure measurements were taken prior to pregnancy as well as at GD15 and GD20. The animals were placed in mouse cages, warmed to 37°C, and wrapped in a surgical towel to minimize stress due to forcible restraint. Two cuffs (occlusion cuff and volume pressure recording cuff) were attached to their tails. Mean arterial blood pressures, diastolic and systolic blood pressure and heart rate readings were measured for each animal (Coda6, Kent Scientific, Torrington, CT). The animals underwent 5 acclimatization cycles and 20 measurement cycles. The reported values are the mean of these 20 cycles.

### Maternal metabolic parameters: Pre-pregnancy measures

Glucose tolerance was determined in CON- and HF-fed dams prior to pregnancy following a 16 hr fast. Animals were given 2 g/kg body weight D-glucose by gavage. Blood was sampled from the saphenous vein at 0, 30, 60 and 120 min. Insulin tolerance tests were carried out following a 16 hr fast. Animals were injected with 1 U/kg of human insulin (Novolin ge, Novo Nordisk, Bagsvared, Denmark) and blood was sampled from the saphenous vein at 0, 30, 60, 120 min. The fasting blood glucose (Pointe Scientific, Canton, MI), serum triglycerides (Pointe Scientific Inc, Canton, MI), non-esterified fatty acids (NEFA; Roche Diagnostics, Indianapolis, IN) and total cholesterol (Pointe Scientific Inc, Canton, MI) were quantified using colorimetric assays according to manufacturers' instructions. Insulin was measured using a high-sensitivity rat insulin ELISA (Crystal Chem Inc, Downers Grove, IL) while leptin was quantified using a direct rat leptin ELISA kit (Crystal Chem Inc, Downers Grove, IL).

### Calculation of HOMA-IR

The homeostatic model of insulin resistance (HOMA-IR) was calculated [58] from fasting insulin and glucose concentrations using the formula HOMA-IR = fasting glucose (mmol/L)×fasting insulin (mU/L)/22.5.

### Immunohistochemistry

Formalin-fixed whole placenta were embedded and sectioned at a thickness of 5 µm. Sections were de-paraffinized in xylene and rehydrated in graded alcohol solutions. Immunohistochemistry (IHC) was performed to determine localization and expression of CD31 (an endothelial cell marker), smooth muscle actin (SMA, a marker of blood vessel maturity), and carbonic anhydrase (CA, a marker of tissue hypoxia). Endogenous peroxidase activity was inhibited using 1% hydrogen peroxide for 10 minutes at RT. Antigen retrieval was achieved by immersing slides in 10 mM citrate buffer at 90°C for 12 minutes. Tissues were blocked using 5% BSA for 10 minutes at RT. Tissues were incubated with anti-CD31 (BD Biosciences Pharmingen, San Diego, CA) or rabbit anti-carbonic anhydrase-IX (1∶500 dilution; Abcam, Cambridge MA) overnight at 4°C in a humidified chamber. The following day, sections were incubated with anti-mouse (CD31) biotinylated secondary antibody (Sigma-Aldrich Canada Ltd., Oakville, ON) or biotinylated goat anti-rabbit (CA) (1∶500 dilution; Vector Labs, Burlingame CA) for 2 hours at RT. Tissues were then exposed to ExtrAvidin® (Sigma-Aldrich Canada Ltd., Oakville, ON) for 1 hour at RT and antibodies were visualized using DAB (Sigma-Aldrich Canada Ltd., Oakville, ON). Tissue was counterstained with Carazzi's Hematoxylin, dehydrated and mounted on coverslips. Slides were imaged using brightfield microscopy.

Blood vessel density and maturity were quantified using the Metamorph integrated morphometry software (Molecular Devices, Downingtown, PA). For the determination of vessel density, a minimum of 4 fields of view per tissue section were used. We quantified sections from 5 individual animals per group. All analysis was carried out in a blinded fashion. Mature blood vessels were characterized as having pericyte coverage (SMA positive vessels) [43]. To quantify the level of tissue hypoxia, the percentage of tissue immunopositive for carbonic anhydrase was measured.

### Western blotting

Whole placental tissue samples were homogenized in a 1∶25 ratio of tissue to homogenization buffer (5 mM HEPES, pH 7.2, 100 mM KCl, 70 mM sucrose, 220 mM mannitol, 1 mM EGTA) with protease inhibitor cocktail tablets (Roche diagnostics, Indianapolis IN). Levels of 4-hydroxynonenal (4-HNE) and nitrotyrosine were quantified using Western blotting, as previously described [59]. Briefly, 20 µg of placental homogenate was separated on a 12.5% SDS-PAGE gel and transferred to nitrocellulose membranes. Membranes were blocked in 5% bovine serum albumin (BSA; Roche, Indianapolis, IN) in Tris buffered saline (TBST: 137 mM NaCl, 2.7 mM KCl, 25 mM Tris-Cl, pH 8.0) supplemented with 0.1% Tween-20 overnight at 4°C. Membranes were then incubated with anti-4-HNE (1∶1000; Abcam Inc, Cambridge, MA) or anti-nitrotyrosine (1∶5000; Millipore, Billerica, MA) in 5% BSA in TBST for 24 hours and washed with TBST before incubation with anti-mouse IgG secondary antibody (1∶5000; GE Healthcare, Mississauga ON) in 5% BSA in TBST. Blots were developed using enhanced chemiluminescence (ECL) (Millipore, Billerica, MA) and densitometric quantification was carried out using ImageJ software (ImageJ, Version 1.37, NIH, Bethesda, MD, USA).

### Measurement of 8-hydroxy-2′-deoxyguanosine (8-OH-2-dG) in urine

8-OH-2-dG, a marker of oxidative DNA damage, was measured in urine samples from GD15 dams using a commercially available EIA kit (Cayman Chemical, Ann Arbor, MI) and normalized to urinary creatinine (Assay Designs, Ann Arbor, MI).

### Measurement of protein carbonyls in isolated mitochondria

Protein carbonyls, a marker of oxidative damage to proteins were measured in mitochondria isolated from whole placental homogenates. Placental tissues were homogenized at a ratio of 1∶5 with homogenization buffer (5 mM HEPES, pH 7.2, 100 mM KCl, 70 mM sucrose, 220 mM mannitol, 1 mM EGTA, 2 mg/mL fatty acid free BSA) plus protease inhibitor cocktail tablets (Roche diagnostics, Indianapolis, IN) for 20 sec using a Polytron homogenizer. These homogenates were rehomogenized manually using a dounce homogenizer (Wheaton, Millville, NJ), and centrifuged (Avanti J-301, Beckman Coulter, Fullerton, CA) at 1200× g for 10 minutes at 4°C. The supernatant was centrifuged at 12,000× g for 10 minutes at 4°C. The pellet was then washed in 1 mL of homogenization buffer without 2 mg/mL BSA, twice. The final pellet was resuspended in 250 µl of homogenization buffer without BSA [60].

Total protein carbonyl content in mitochondrially enriched fractions prepared from GD15 placental homogenates was determined using a commercially available kit (Oxyblot Protein Oxidation Detection Kit, Chemicon International, Inc, Temecula, CA, USA), according to the manufacturer's instructions.

### Statistical Evaluation

All statistical analysis was performed using GraphPad v4.0 for Windows (GraphPad Software, San Diego, CA). Outcome measures (continuous variables) between CON and HF groups were compared using Student's t-test (alpha = 0.05) and categorical variables were compared using Fisher's exact test. Each dam represents a single statistical unit. All data were tested for normality and homogeneity of variance.

## Supporting Information

Figure S1 **Weight gain prior to pregnancy.** Weight gain of CON-fed (triangle) vs. HF–fed (black circle) dams prior to pregnancy. Values represent mean ± SEM; n≥29.
(TIF)

Figure S2 **Gestational blood pressure changes.** Mean arterial blood pressure changes were measured 7 days prior to mating (indicated at gestation day −7) in CON-fed (open triangle) vs. HF–fed (black circle) dams as well as at GD15 and GD20. The initiation of gestation is indicated by the arrow. Blood pressure values represent mean ± SEM; n≥17; *p<0.05.
(TIF)

Figure S3 **Nitrotyrosine damage in the placenta of obese dams is not significantly increased at GD15.** A. 10 µg of whole placental homogenate was separated on a 12.5% SDS-PAGE and subjected to Western blot analysis. The average content of nitrotyrosine was normalized to total protein (using Ponceau-S staining) in CON-fed and HF-fed dams. B. Representative lanes containing 10 µg placental homogenate developed using an antibody directed towards nitrotyrosine. Values represent mean ± SEM; n = 15 for CON and n = 10 for HF-fed dams.
(TIF)

Table S1 **Nutrient composition of rodent diets.**
(DOCX)
