# Supplementary figures and images for: Adverse Fetal and Neonatal Outcomes Associated with a Life-Long High Fat Diet: Role of Altered Development of the Placental Vasculature
Source: PLoS One. 2012 Mar 19;7(3):e33370. doi: 10.1371/journal.pone.0033370 (PMC3307735; doi:10.1371/journal.pone.0033370)

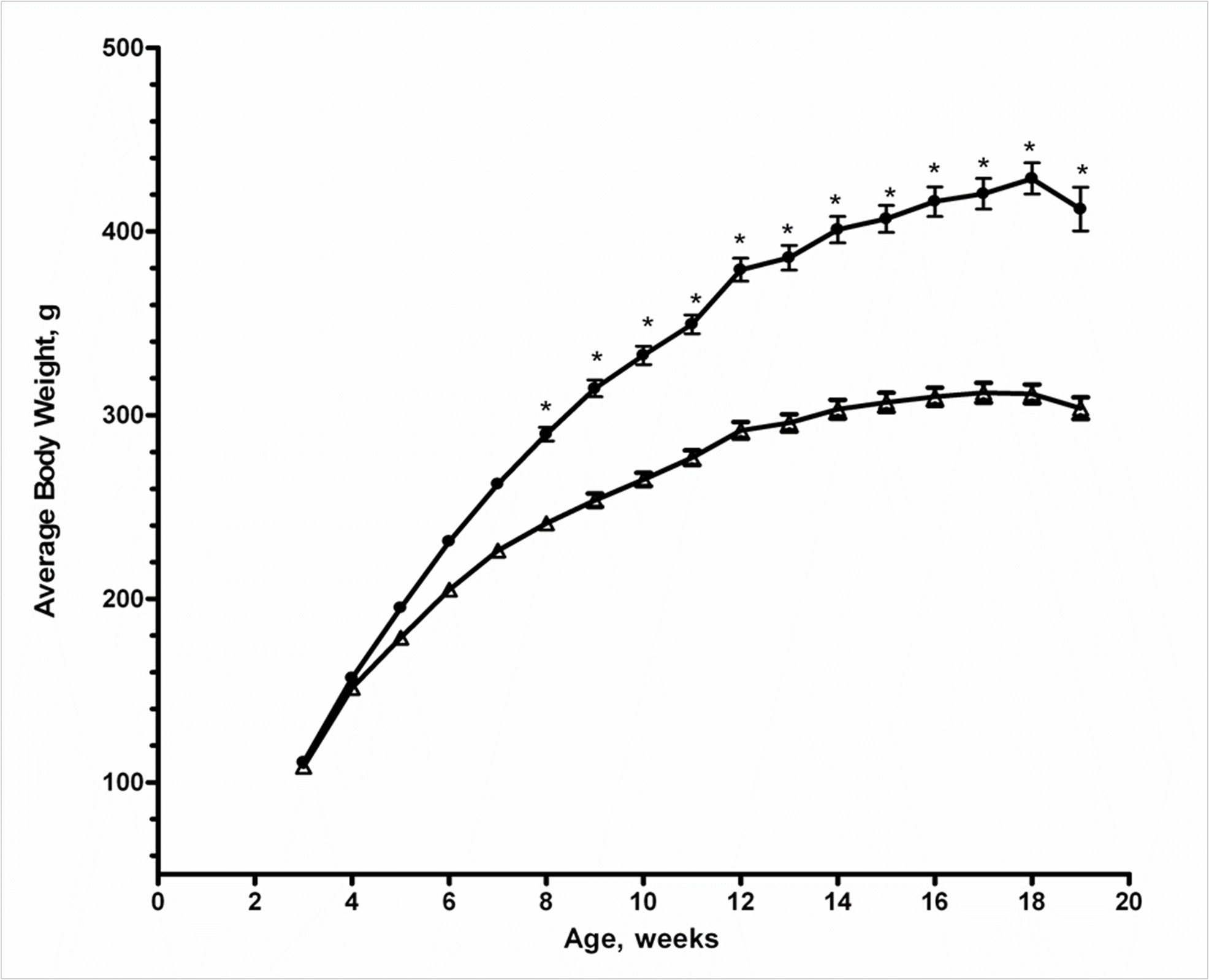

Supplement: Figure S1 — Weight gain prior to pregnancy. Weight gain of CON-fed (triangle) vs. HF–fed (black circle) dams prior to pregnancy. Values represent mean ± SEM; n≥29. (TIF) [file pone.0033370.s001.tif]

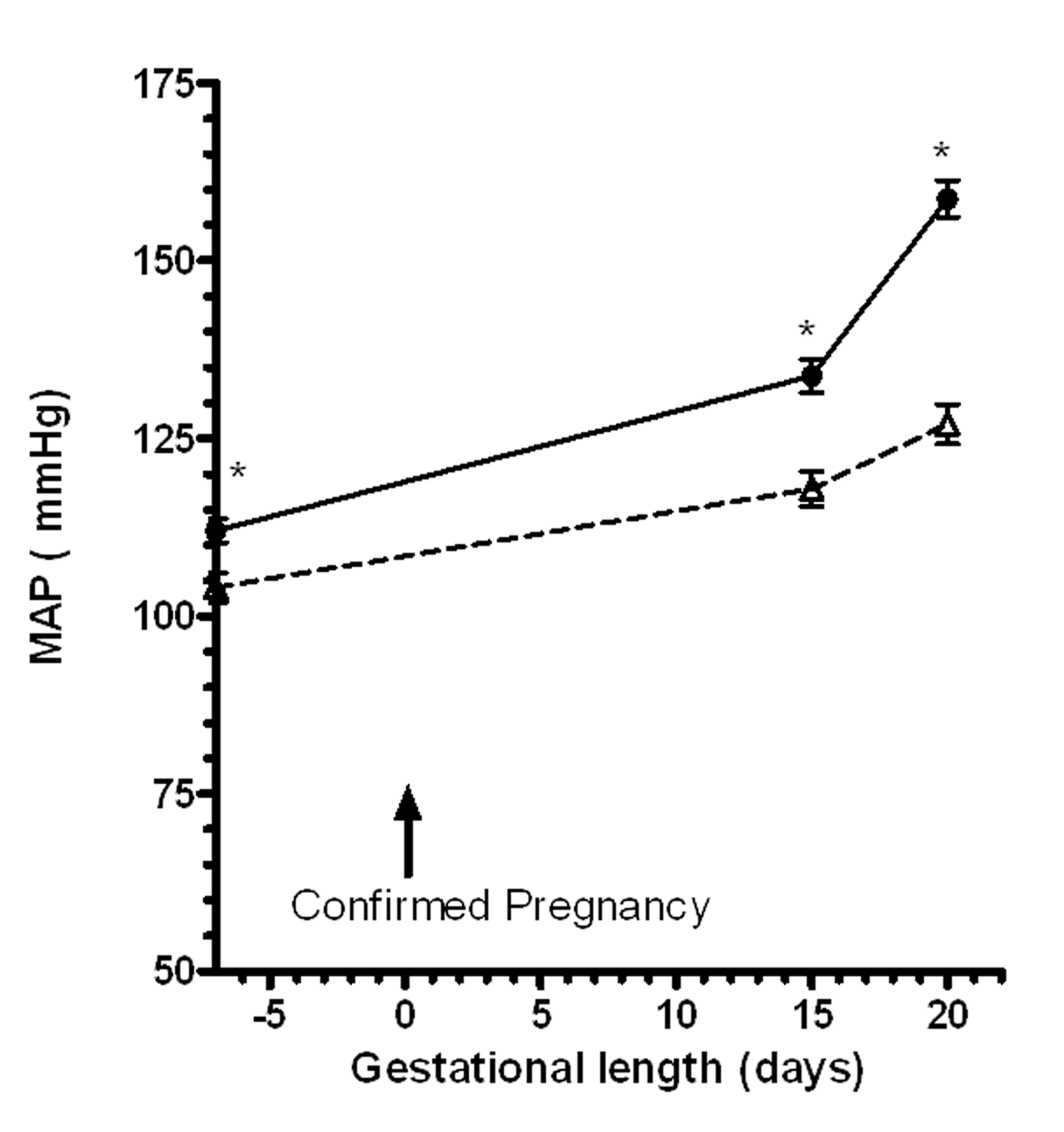

Supplement: Figure S2 — Gestational blood pressure changes. Mean arterial blood pressure changes were measured 7 days prior to mating (indicated at gestation day −7) in CON-fed (open triangle) vs. HF–fed (black circle) dams as well as at GD15 and GD20. The initiation of gestation is indicated by the arrow. Blood pressure values represent mean ± SEM; n≥17; *p<0.05. (TIF) [file pone.0033370.s002.tif]

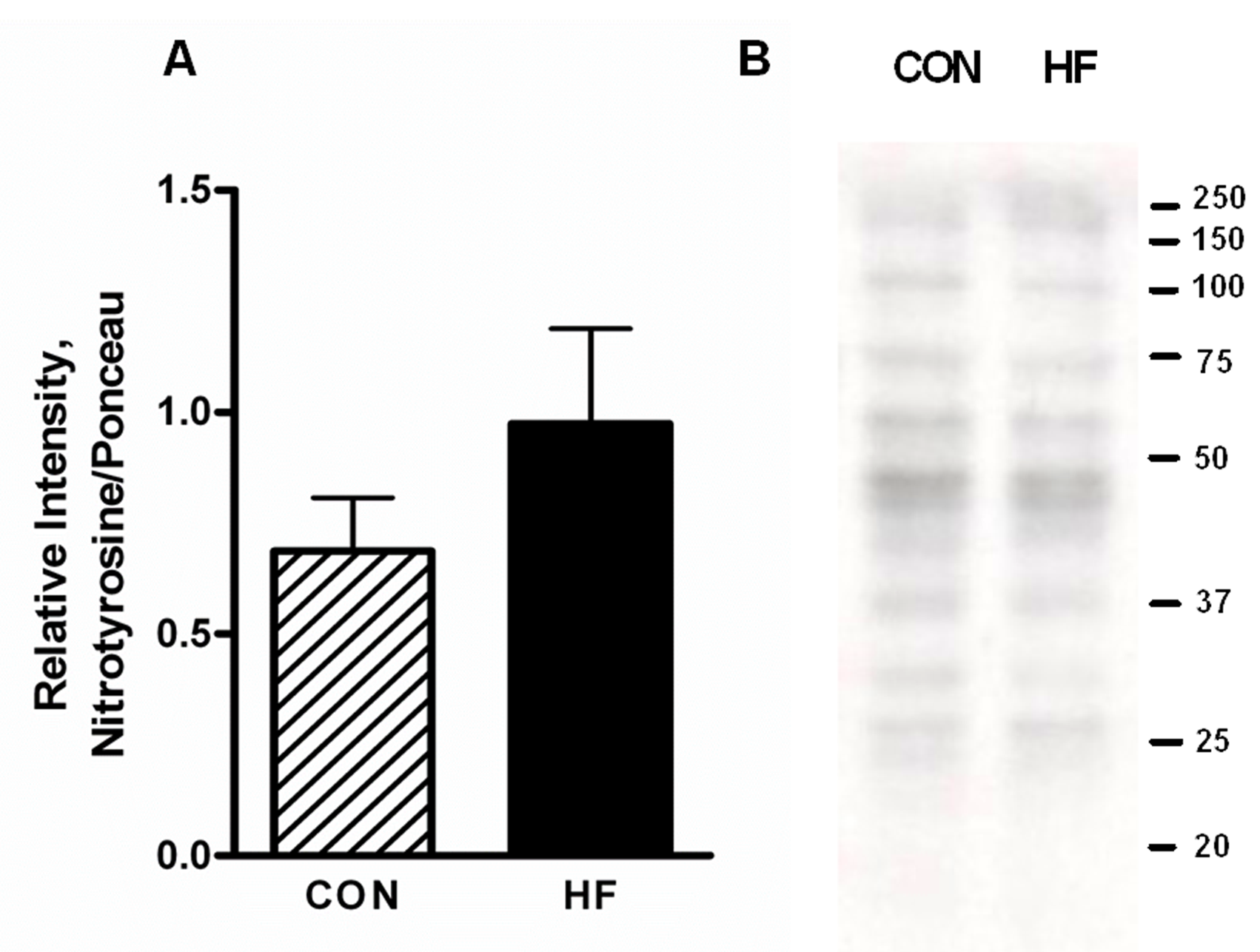

Supplement: Figure S3 — Nitrotyrosine damage in the placenta of obese dams is not significantly increased at GD15. A. 10 µg of whole placental homogenate was separated on a 12.5% SDS-PAGE and subjected to Western blot analysis. The average content of nitrotyrosine was normalized to total protein (using Ponceau-S staining) in CON-fed and HF-fed dams. B. Representative lanes containing 10 µg placental homogenate developed using an antibody directed towards nitrotyrosine. Values represent mean ± SEM; n = 15 for CON and n = 10 for HF-fed dams. (TIF) [file pone.0033370.s003.tif]
