# Supplementary material for: Adverse Fetal and Neonatal Outcomes Associated with a Life-Long High Fat Diet: Role of Altered Development of the Placental Vasculature
Source: PLoS One. 2012 Mar 19;7(3):e33370. doi: 10.1371/journal.pone.0033370 (PMC3307735; doi:10.1371/journal.pone.0033370)
Supplement: Table S1 — Nutrient composition of rodent diets. (DOCX) [file pone.0033370.s004.docx]

**Supplemental Table 1. Macronutrient composition of CON and HF diets.**

|  | **Diet composition, % kcal** | |
| --- | --- | --- |
|  | **CON** | **HF** |
| **Protein** | 29 | 20 |
| **Carbohydrate** | 54 | 35 |
| **Fat** | 17 | 45 |
